# Photoelectrocatalytic hydrogen evolution reaction stimulated by the surface plasmon resonance effect of copper and silver surrounded with MoS_2_†

**DOI:** 10.1039/d3ra04357f

**Published:** 2023-08-23

**Authors:** Yuanyuan Tian, Chengnan Qi, Ruihua Zhou, Dan Li, Tao Han

**Affiliations:** a College of Materials Industry, Shanxi College of Technology Shuozhou Shanxi Province 036000 China tyy20220418@163.com

## Abstract

Developing plasmonic metal-based photocatalysts can improve the photoelectrocatalytic hydrogen evolution reaction (HER) and overcome the limitations of semiconductor-based photocatalysts. A MoS_2_@Ag–Cu foam composite electrode was fabricated on a copper foam and employed for photoelectrocatalytic HER. The optical behavior and photoelectrocatalytic HER test results indicate that the surface plasmon resonance effect of copper and silver is the primary source of light absorption. Additionally, molybdenum sulfide was employed as a hot-electron trap to capture the energetic electrons generated from copper and silver, thereby promoting the hydrogen evolution reaction. Its binder-free electrode exhibits the better HER performance and excellent stability. Low-cost plasmonic metals, copper and silver, were used as the source of photocatalysis, providing a novel perspective for enhancing photoelectrocatalytic HER performance.

## Abstract 2

A self-supported photocathode exhibits superior stability and enhanced photoelectrocatalytic performance. The band structure of MoS_2_ was engineered to enable efficient capture and utilization of photoelectrons generated from the SPR effect.

## Graphical abstract

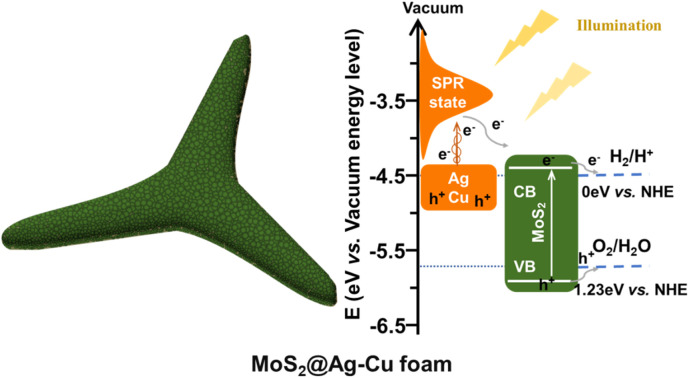

## Introduction

Photoelectrocatalytic HER has been identified as one of the best ways to address the energy crisis and environmental pollution.^1^ The first-generation semiconductor-based photoelectrocatalyst materials were proposed by scientists Fujishima and Honda in the 1970s.^2^ In the following decades, they were continuously utilized for enhancing photoelectrocatalytic reactions through modification^3–6^ and decoration.^7–11^ However, low electric conductivity and narrow window for light absorption^12,13^ became fatal flaws that limited the HER performance. In recent years, plasmonic metal-based materials, such as precious metal Au,^14–17^ have been considered a novel and reliable means of boosting the photoelectrocatalytic HER.^18,19^ Research has shown that plasmonic metals can effectively improve photoelectrocatalytic performance due to their three advantages: the antenna effect, adjustable resonance energy, and ultra-sensitive sensing.^20–22^ Thus, plasmonic metals are expected to become the next generation of photocatalysts. Besides, metals with high conductivity promote electron transfer and rapid electrocatalytic reactions.^23^ However, the cost of photoelectrodes has increased due to the use of precious metals in the preparation process. Therefore, it is imperative to utilize low-cost plasmonic metals such as copper and silver for efficient light harvesting and hot electron generation. The photogenerated carriers resulting from plasmon resonance effects are more prone to recombination compared to those in semiconductor-based materials.^24,25^ The most significant subject now is how to capture and utilize the hot electrons generated for stimulating HER.

Molybdenum sulfide exhibits a free energy of adsorbed H comparable to that of Pt,^26–28^ which is considered the best HER catalyst and can replace noble catalysts. Furthermore, amorphous molybdenum sulfide has been demonstrated to exhibit superior catalytic performance due to the presence of catalytically active sites located at lattice defects.^29–31^ The two-dimensional layered molybdenum sulfide, with a band gap of 1.8 eV, can generate photo-induced carriers under visible light illumination.^32,33^ The appropriate band structure of molybdenum sulfide can trap the hot electrons^34^ originated from surface plasmon resonance effect, which synergistically participate in photoelectrocatalytic HER.

In this paper, a hierarchical porous copper foam (Cu foam) was used as a conductive substrate. Ag nano-layers and amorphous molybdenum sulfide were successively grown *in situ* on the Cu foam by means of wet chemical and electrochemical deposition methods (MoS_2_@Ag–Cu foam). The prepared electrodes were characterized by scanning electron microscopy (SEM), X-ray diffraction (XRD), X-ray photoelectron spectroscopy (XPS), UV-visible absorption spectroscopy, and photoluminescence spectroscopy (PL). The results of the photoelectrocatalytic HER test indicate that the MoS_2_@Ag–Cu foam electrode exhibits the strongest photoelectrocatalytic performance, with a photo response current density (Δ*J*) 2.88 times higher than that of Cu foam and 1.83 times higher than that of Ag–Cu foam. Additionally, MoS_2_@Ag–Cu foam electrode demonstrated superb electrochemical stability. It offers a new perspective for the field of photoelectric HER.

## Experimental

### Materials

Sulfuric acid, hydrochloric acid, ethanol, silver nitrate, potassium chloride, ammonium hydroxide solution, ammonium tetrathromolybdate and sodium sulfide nonahydrate were purchased from Macklin. Copper foam (200 × 100 × 1.5 mm, 99.8% purity) was obtained from ZhongNuo Advanced Material (Beijing) Technology Co., Ltd. Deionized water (DI water) (resistivity > 18.2 MΩ cm^−1^) was prepared by a pure water equipment (TTL-6B). All chemical reagents were used as received.

### Preparation of the composite electrode

**Scheme 1:**
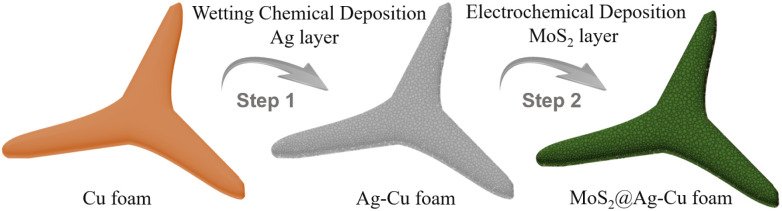
Schematic illustration of the synthesis process of MoS_2_@Ag–Cu foam.

As shown in Scheme 1, MoS_2_@Ag–Cu foam electrode was synthesized on the copper foam with 3D hierarchical porous structure through the following two-step procedure.

Step 1: Ag nano-layers grown *in situ* on the Cu foam by wetting chemical deposition method (Ag–Cu foam).

Firstly, Cu foam was cut into rectangle with the area of 2 × 4 cm, and it was washed by acetone, ethanol, hydrochloric acid, and deionized water respectively. After that, 0.75 g AgNO_3_ was dissolved in the mixture of 12 mL NH_3_·H_2_O, 8 mL CH_2_O and 100 mL deionized waster. Next, the cleaned Cu foam was immersed into the solution with stirring for 15 minutes. At last, the grown *in situ* Ag nano-layer on Cu foam was washed by deionized water and dried in the N_2_ stream. The as-obtained electrode was named Ag–Cu foam.

Step 2: molybdenum sulfide grown *in situ* on the Ag–Cu foam by electrochemical deposition method (MoS_2_@Ag–Cu foam).

Molybdenum sulfide coated on Ag–Cu foam *via* a facile electrochemical deposition method described by previous work.^35^ Specifically, 0.104 g (NH_4_)_2_MoS_4_, 1.5 g KCl and 0.096 g Na_2_S·9H_2_O were dissolved in the 200 mL deionized waster. Above solution was used as electrolyte, and carbon rod and saturated calomel electrode (SCE) were used as counter and reference electrodes, respectively. The Ag–Cu foam electrode was directly used as working electrode. The electrodeposition mood was set to chronopotentiometry with 0.075 mA for 10 minutes. After that, the as-obtained electrode was washed by deionized water and dried in the N_2_ stream, and it was denoted as MoS_2_@Ag–Cu foam.

### Characterization

Scanning electron microscopy (SEM, JEOL JSM-7800F) was employed to investigate the morphology and microstructure of the as-prepared electrodes. A small piece of electrodes can be directly employed as the sample for characterization of SEM and energy dispersive spectrum. X-ray diffraction (XRD) patterns were taken on an X-ray diffractometer (2500VB2+PC, Japan) with Cu Kα radiation. X-ray photoelectron spectroscopy (XPS) analysis was detected on an Thermo ESCALAB 250. The optical behaviours were detected by ultraviolet-visible spectrophotometer (Shimadzu UV3600, Japan). Photoluminescence measurements (PL) were examined on a spectrophotometer (Hitachi F-7000) at an excitation light of 410 nm. The electrodes prepared were directly utilized as the detected samples.

### Photoelectrochemical measurements

Photoelectrocatalytic HER measurements were conducted using a standard three-electrode system in 0.5 M H_2_SO_4_ aqueous solution on the electrochemical workstation (CHI, 760E). The as-prepared electrodes were cut into pieces with a surface area of 0.25 cm^2^ and directly employed as the working electrode. A saturated calomel electrode (SCE) and a carbon rod were employed as the reference and counter electrode. The electrocatalytic activity was evaluated by recording linear sweep voltammetry (LSV) with a scan rate of 5 mV s^−1^. The potentials in the LSV curves were IR-corrected based on the ohmic resistance of the solution. The electrochemical impedance spectroscopy (EIS) measurements were performed at −0.2 V *vs.* RHE over potential in frequency from 10^−1^ to 10^4^ Hz. The cyclic voltammetric method was taken to evaluate the electrochemical double layer capacitance (*C*_dl_) in the non-Faraday region from 0 to 0.1 V *vs.* RHE with the scan rates are 150, 120, 100, 80, and 60 mV s^−1^, respectively. The visible light source was simulated by a 300 W Xe arc lamp (CEL-HXF300) assembled with an AM 1.5 filter.

## Result and discussion

### Structure characterization

**Fig. 1:**
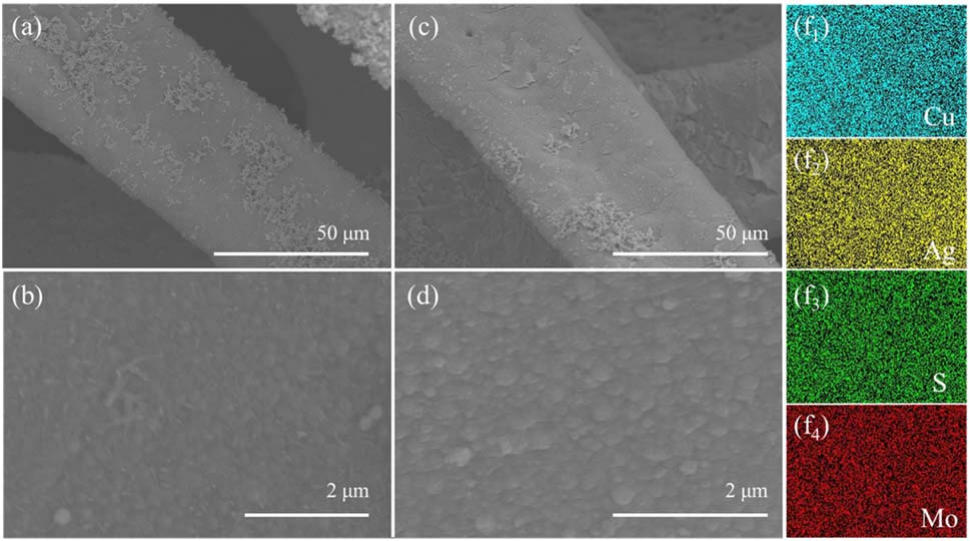
SEM images of (a) Ag–Cu foam and (c) MoS_2_@Ag–Cu foam, higher-magnification of SEM images of (b) Ag–Cu foam and (d) MoS_2_@Ag–Cu foam respectively. (f_1–4_) Corresponding EDS mapping images of MoS_2_@Ag–Cu foam.

The morphologies of Ag–Cu foam and MoS_2_@Ag–Cu foam were examined *via* SEM analysis, as illustrated in Fig. 1. As observed, the Cu foam frame was coated with a layer of Ag nanoparticles (Fig. 1a and b), while still maintaining its initial hierarchical porous structure (Fig. S1a†) after the wetting chemical deposition process. As depicted in Fig. 1c, the skeletons of the Cu foam are uniformly coated with composite catalysts. A higher-magnification SEM image (Fig. 1d) revealed that the diameter of nanoparticles located on Cu foam increased following molybdenum sulfide coating. Furthermore, the corresponding elemental mapping images shown that Mo, S, and Ag elements were uniformly spatially distributed on the Cu foam (Fig. 1f_1–4_), which suggested that the MoS_2_@Ag–Cu foam composite electrode can be synthesized through the two-step procedure. For comparison, MoS_2_@Cu foam were prepared by similar method, as shown in the Fig. S1b and c.†

**Fig. 2:**
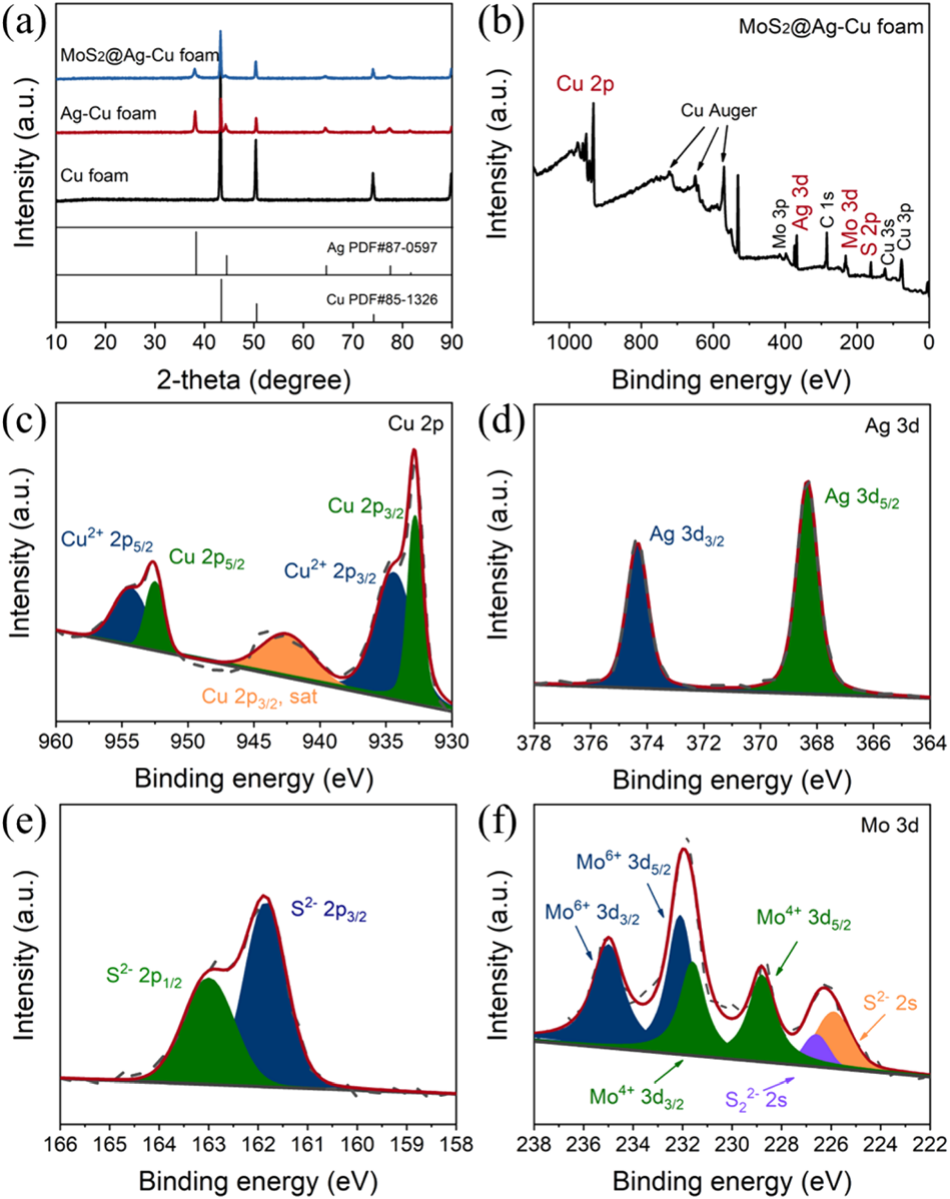
(a) XRD patterns of the Cu foam, Ag–Cu foam, and MoS_2_@Ag–Cu foam. XPS spectrum of the MoS_2_@Ag–Cu foam, (b) survey spectra and corresponding high-resolution XPS (HRXPS) spectrum of (c) Cu 2p, (d) Ag 3d, (e) S 2p, and (f) Mo 3d.

The composition and structure of the synthesized electrodes were also characterized using X-ray diffraction technique, as illustrated in Fig. 2a. The peaks observed at 43.3°, 50.4° and 74.1° can be attributed to (111), (200) and (220) crystallographic planes of copper, respectively, as indicated by JCPDS card no. 85-1326. The peaks identified at 38.1°, 44.3°, 64.4°, 77.4°, and 81.5° were due to (111), (200), (220), (311) and (222) planes of Ag, respectively (JCPDS card no. 87-0597). The diffraction peaks of MoS_2_ were not obviously sharp due to the amorphous nature of the as-prepared molybdenum sulfide.^36^ It should be noted that the diffraction peak of MoS_2_@Ag–Cu foam electrode was significantly attenuated compared to that of Ag–Cu foam electrode, which can be attributed to the amorphous molybdenum sulfide coating covering the diffraction signal of copper and silver.

The chemical states of elements in MoS_2_@Ag–Cu foam was analyzed in detail by XPS, as depicted in Fig. 2b–f. The XPS survey spectra revealed the presence of major elements including Cu, Ag, S, and Mo (Fig. 2b). In Fig. 2c, two prominent peaks observed at 952.5 eV and 932.8 eV corresponded to Cu 2p_1/2_ and Cu 2p_3/2_ orbitals of Cu, respectively. The peaks at 954.4 eV and 934.4 eV were ascribed to Cu^2+^ 2p_1/2_ and Cu^2+^ 2p_3/2_ of CuO, respectively, which resulted from its oxidation in the air.^37^ The peaks observed at 374.4 eV and 368.3 eV can be attributed to the Ag 3d_3/2_ and Ag 3d_5/2_ orbitals of silver, respectively, as depicted in Fig. 2d. Meanwhile, the peaks at 163.0 eV and 161.8 eV are assigned to the S^2−^ 2p_1/2_ and S^2−^ 2 p_3/2_ orbitals of sulfide, respectively (Fig. 2e). In Fig. 2f, the Mo^4+^ 3d_3/2_ and Mo^4+^ 3d_5/2_ peaks were detected at 231.6 eV and 228.8 eV, respectively. The peaks located at 235.0 eV and 232.1 eV were attributed to Mo^6+^ 3d_3/2_ and Mo^6+^ 3d_5/2_, respectively, because of the reoxidation process of MoS_2_. Additionally, two peaks appearing at 226.6 eV and 225.9 eV can be assigned to S_2_^2−^ 2s and S^2−^ 2s orbitals of amorphous MoS_2_.^36^ The SEM, XRD and XPS analyses provided comprehensive evidence that the Cu foam substrate was effectively coated with a bilayer of silver nanoparticles and molybdenum sulfide.

### Optical property

**Fig. 3:**
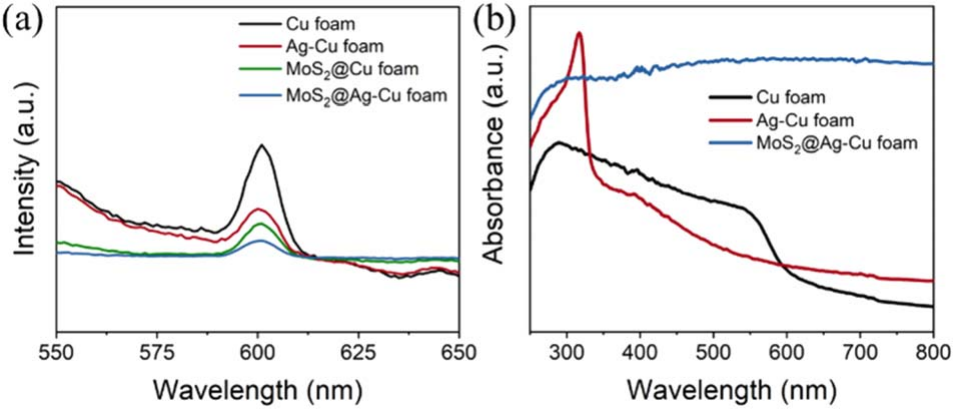
(a) Photoluminescence spectra, and (b) UV-visible absorption spectra of Cu foam, Ag–Cu foam, and MoS_2_@Ag–Cu foam.

The rate of photoinduced carrier recombination is a crucial factor that impacts the photoelectrocatalytic performance, which can be evaluated through photoluminescence (PL) emission spectra analysis. Fig. 3a displayed the normalized PL emission spectra of the samples obtained, such as pure Cu foam, Ag–Cu foam, MoS_2_@Cu foam, and MoS_2_@Ag–Cu foam, through fluorescence spectroscopy with an excitation wavelength of 410 nm. Evidently, Cu foam and Ag–Cu foam, without MoS_2_ layer, exhibited the most prominent diffraction signal at 600 nm, indicating that it can generate a significant number of photogenerated carriers originated from the plasmon resonance effect of copper and undergoes rapid recombination.^8,15^ Significantly weaker emission signals were observed from MoS_2_@Ag–Cu foam and MoS_2_@Cu foam, both with a layer of MoS_2_, compared to those from Cu foam and Ag–Cu foam. This suggested that the presence of MoS_2_ can effectively trap hot carriers and hinder their recombination. The UV-vis absorption spectra of the obtained samples was analyzed, as illustrated in Fig. 3b. The absorption peak at 580 nm observed in the pristine Cu foam electrode was attributed to the surface plasmon resonance (SPR) absorption of copper.^38^ In comparison with Cu foam, the additional absorption in Ag–Cu foam at wavelength shorter than 350 nm was ascribed to the SPR absorption of silver.^39^ More importantly, the light absorption for MoS_2_@Ag–Cu foam was significantly extended to visible absorption region due to the narrow band gap of MoS_2_.^40,41^

**Fig. 4:**
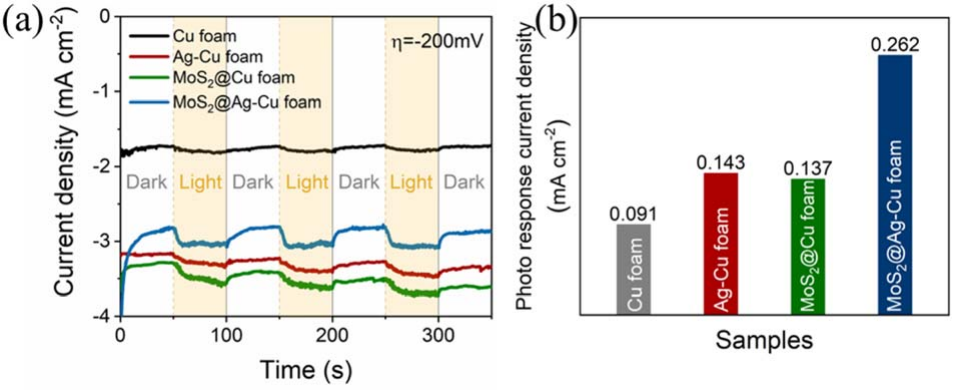
(a) On–off *J*–*t* curves of Cu foam, Ag–Cu foam, MoS_2_@Cu foam, and MoS_2_@Ag–Cu foam. The potential bias is −200 mV. (b) Corresponding photo response current density of the as prepared samples was calculated between 200 s and 250 s for (a).

To demonstrate the effective photoelectrocatalytic properties facilitated by the SPR effect and molybdenum sulfide enhancement, we conducted a comparative analysis of the photo response current density for the obtained samples. As illustrated in Fig. 4a, the photocurrent chronoamperometry was performed with chopped light irradiation at potential of −200 mV. The photoresponsivity of Cu foam was evident, while that of Ag–Cu foam electrode exhibited even higher enhancement, and their photo response current densities were 0.091 and 0.143 mA cm^−2^, respectively. After applying a molybdenum sulfide trap, the photo response current density of MoS_2_@Ag–Cu foam electrode increased twofold compared to that of Ag–Cu foam, as illustrated in Fig. 4b. On the contrary, MoS_2_/FTO electrode, in the absence of plasmonic metal, exhibited a negligible photo response current density under identical conditions (Fig. S2†).

### The stability of the electrode

**Fig. 5:**
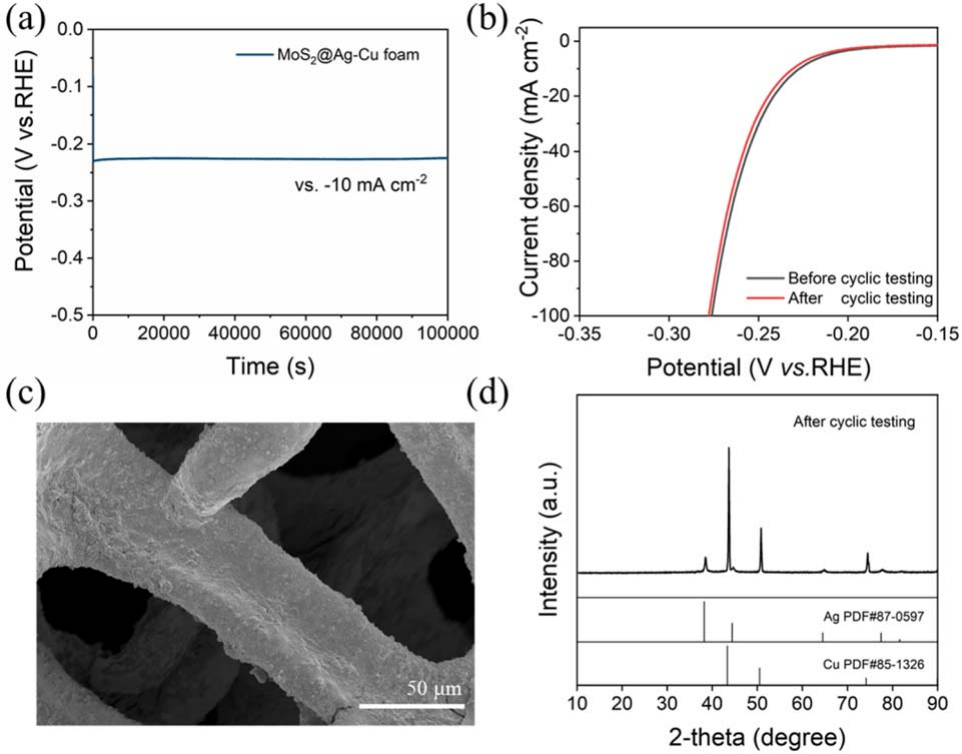
(a) Chronopotentiometry curve of MoS_2_@Ag–Cu foam at a constant current density of 10 mA cm^−2^ in 0.5 M H_2_SO_4_. (b) LSV curves of MoS_2_@Ag–Cu foam before and after cyclic testing. (c) SEM images and (d) XRD pattern of MoS_2_@Ag–Cu foam after cyclic testing. The cyclic tests were performed for 1000 cycles, ranging from 0 to −0.3 V *vs.* RHE at a scan rate of 10 mV s^−1^.

The superior stability of MoS_2_@Ag–Cu foam composite electrode was confirmed *via* electrochemical and physical techniques. As shown in Fig. 5a, the composite electrode exhibited exceptional potential stability over 28 hours at 10 mA cm^−2^. Fig. 5b showed that the LSV curve after cyclic testing exhibited a negligible decline compared to the pristine curve. The SEM image (Fig. 5c), XRD pattern (Fig. 5d), and EDS mapping images (Fig. S3†) of MoS_2_@Ag–Cu foam after long-term cyclic testing indicated structural and morphological stability.

### Mechanism analysis

**Fig. 6:**
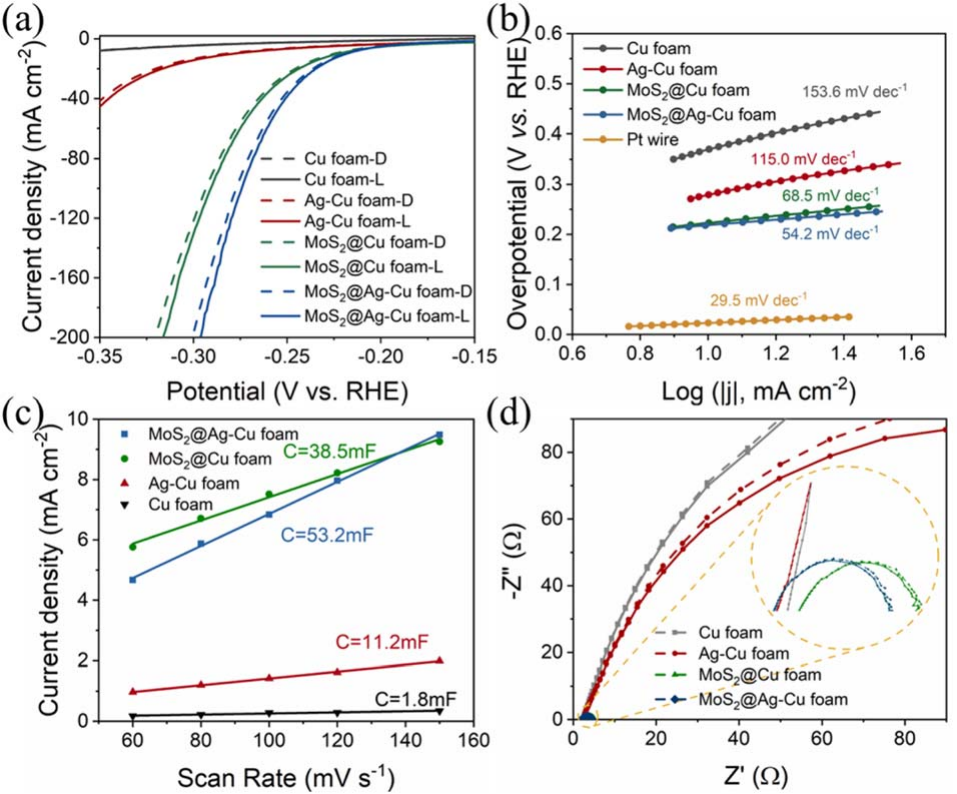
(a) LSV curves of the samples prepared. Control experiments are conducted in the dark (indicated by dotted line) and under illumination (indicated by solid line), respectively. (b) Tafel plots derived from the LSV curves. (c) Estimation of *C*_dl_ by plotting the current density variation *vs.* scan rate to fit a linear regression. (d) EIS studies at overpotential of *η* = −200 mV *vs.* RHE, inset images are corresponding enlargement.

The electrochemical measurements were analyzed to elucidate the mechanism underlying the enhancement of photoelectrocatalytic hydrogen evolution. The electrochemical activity measurements of MoS_2_@Ag–Cu foam for HER in 0.5 M H_2_SO_4_ solution were carried out using a typical three-electrode cell setup in the dark and under illumination, as depicted in Fig. 6a. For comparison, the HER activities of bare Cu foam, Ag–Cu foam, and MoS_2_@Cu foam were investigated. The LSV curves of the composite electrodes exhibited improved HER performance under illuminated conditions compared to those observed in the absence of light. And the composite electrodes coated with molybdenum sulfide indicated enhanced electrocatalytic activity towards HER. The MoS_2_@Ag–Cu foam electrode exhibited an overpotential of 218 mV at current density of 10 mA cm^−2^, surpassing those of bare Cu foam, Ag–Cu foam, and MoS_2_@Cu foam, whether in the dark or under illumination. The specific overpotential values at 10 mA cm^−2^ and 50 mA cm^−2^ under illumination were presented in Table S1.†

The Tafel slope, obtained from the LSV curves of prepared electrodes, serves as an indicator of the intrinsic catalytic activity. As is shown in Fig. 6b, the Tafel slope of MoS_2_@Ag–Cu foam (54.2 mV dec^−1^) was comparable to that of MoS_2_@Cu foam (68.5 mV dec^−1^), yet significantly lower than that of Ag–Cu foam (115 mV dec^−1^) and pure Cu foam (153 mV dec^−1^). This suggested that the outer layer of molybdenum sulfide can serve as an electron mediator between plasmonic meatal and H^+^ in aqueous solution, stimulating rapid kinetics for HER.

The enhanced photoelectrocatalytic performance of MoS_2_@Ag–Cu foam was also attributed to its higher electrochemically active surface area (ECSA). The estimation of the ECSA at the solid–liquid interface can be achieved through the measurement of electrochemical double-layer capacitance (EDLC) *via* CV test.^42^ The EDLC values were determined by calculating the slope of the linear regression analysis of the capacitive current plotted against scan rate (Fig. S4†). As shown in Fig. 6c, the EDLC value of MoS_2_@Ag–Cu foam (53.2 mF cm^−2^) was ∼1.4 times than that of MoS_2_@Cu foam (38.5 mF cm^−2^), ∼4.8 times than that of Ag–Cu foam (11.2 mF cm^−2^) and ∼30 times than that of Cu foam (1.8 mF cm^−2^). This indicated that the MoS_2_@Ag–Cu foam composite electrode possessed the highest ECSA. The values of Tafel slop and ECSA were exhibited in Table S1.†

The Nyquist plot was generated through EIS analysis to evaluate the surface kinetics of the electrodes prepared, as illustrated in Fig. 6d. The diameter of the fitted semicircle represented the charge transfer resistance (*R*_ct_), which denotes the resistance encountered during electron transport. The Nyquist plots of the electrodes with layer of molybdenum sulfide (MoS_2_@Ag–Cu foam and MoS_2_@Cu foam) exhibited a narrower semicircle diameter than that of the other electrodes. The as prepared electrodes displayed the smaller *R*_ct_ values under illumination, especially for electrode with silver nano-layer.

**Scheme 2:**
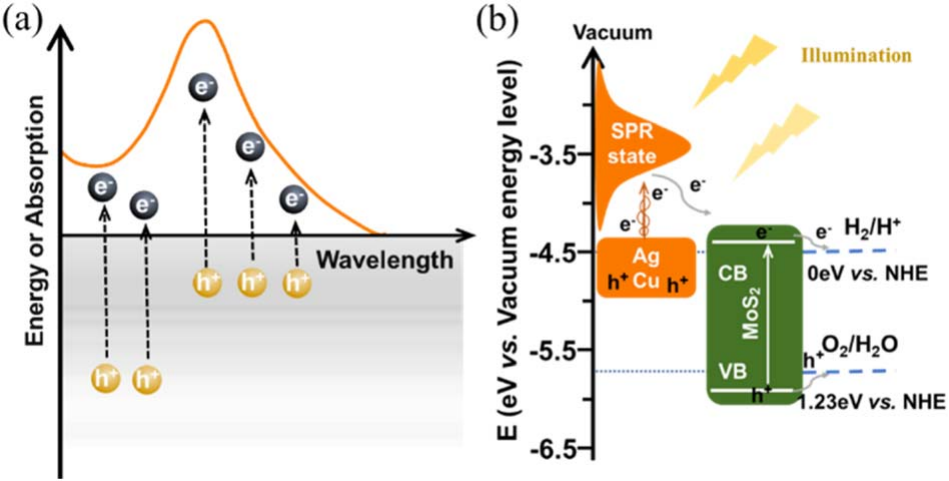
The schematic illustration of (a) hot carriers generation and the corresponding absorption spectrum of plasmonic metals,^34^ and (b) the energy band alignment for charge transfer in MoS_2_@Ag–Cu hybrids catalyst.^38,43^

As illustrated in Scheme 2a, upon exposure to resonant optical radiation, the plasmonic metal exhibited oscillation of its free electrons within the conductive band. Then the hot electron–hole pairs were generated *via* non-radiation attenuated Landau damping within a time frame of 1 to 100 fs.^44^ The plasmon-induced electric field promotes the transition of electrons from occupied states to the higher energy SPR states.^45,46^ Unfortunately, the photoelectrocatalytic performance of pure plasmonic metals is severely limited by their high recombination probability for photogenerated carriers (shown in Fig. 3a) and sluggish kinetics for HER (shown in Fig. 6). For MoS_2_@Ag–Cu hybrids catalyst, on the one hand, silver and copper metals produced a huge number of hot electrons through the SPR effect. On the other hand, the band structure of molybdenum sulfide has been engineered to enable efficient capture and utilization of photoelectrons generated from the SPR effect for stimulating HER reaction, as depicted in Scheme 2b.

## Conclusions

In conclusion, the self-supported MoS_2_@Ag–Cu foam electrode was successfully fabricated as a photocathode through a two-step procedure. The enhanced performance of the MoS_2_@Ag–Cu foam photocathode originated from surface plasmon resonance (SPR) effect of silver and copper, which served as primary optical absorbers. Molybdenum sulfide acted as a hot electron trap, enhancing the separation efficiency of photogenerated carriers and expediting rapid kinetics, to promote hydrogen evolution reaction. Besides, the superior stability of composite electrode is ascribed to the *in situ* growth method employed for its preparation. The development of low-cost plasmonic metal-based materials, the next generation photocatalysts, is key to breaking through the bottleneck of photoelectrocatalytic performance improvement.

## Author contributions

Yuanyuan Tian: conceptualization, methodology, supervision, investigation, funding acquisition, writing-original draft, and writing-review & editing. Chengnan Qi: investigation, formal analysis, data curation, and writing-original draft. Ruihua Zhou, Dan Li: methodology and resources. Tao Han: resources, project administration, and supervision.

## Conflicts of interest

There are no conflicts to declare.

## Supplementary Material

RA-013-D3RA04357F-s001
