# Supplementary material for: Photoelectrocatalytic hydrogen evolution reaction stimulated by the surface plasmon resonance effect of copper and silver surrounded with MoS2
Source: RSC Adv. 2023 Aug 23;13(36):25246–52. doi: 10.1039/d3ra04357f (PMC10445329; doi:10.1039/d3ra04357f)
Supplement: RA-013-D3RA04357F-s001 [file RA-013-D3RA04357F-s001.pdf]

Electronic Supplementary Material (ESI) for RSC Advances.
This journal is © The Royal Society of Chemistry 2023

Supporting information

Photoelectrocatalytic Hydrogen Evolution Reaction Stimulated by Surface Plasmon Resonance Effect of Copper and Silver Surrounded with MoS_2

Yuanyuan Tian, Chengnan Qi, Ruihua Zhou, Dan Li, Tao Han*

College of Materials Industry, Shanxi College of Technology, Shuozhou Shanxi Province, 036000, China.

Correspondence and requests for materials should be addressed to Yuanyuan Tian (email: tyy20220418@163.com)

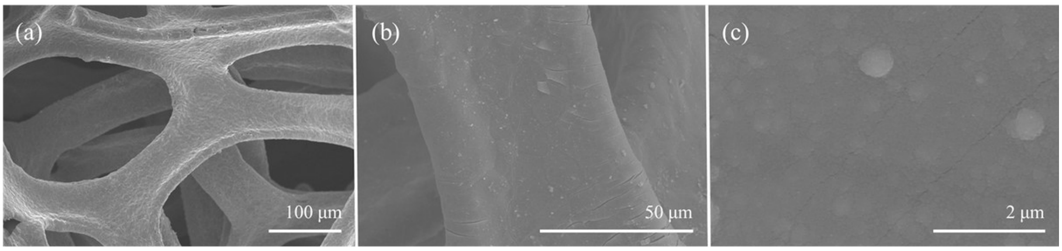

Figure S1. SEM images of (a) Cu foam, (b) MoS_2@Cu foam, (c) corresponding higher-magnification of MoS_2@Cu foam.

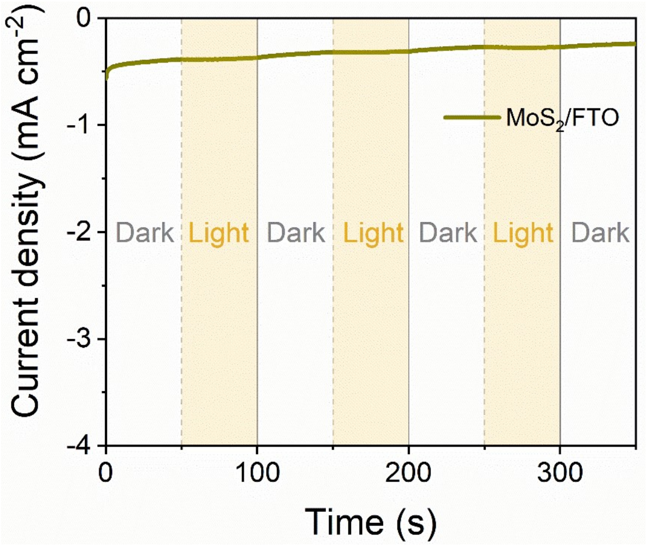

Figure S2. On-off J−t curves of MoS_2/FTO. The potential bias is -200mV.

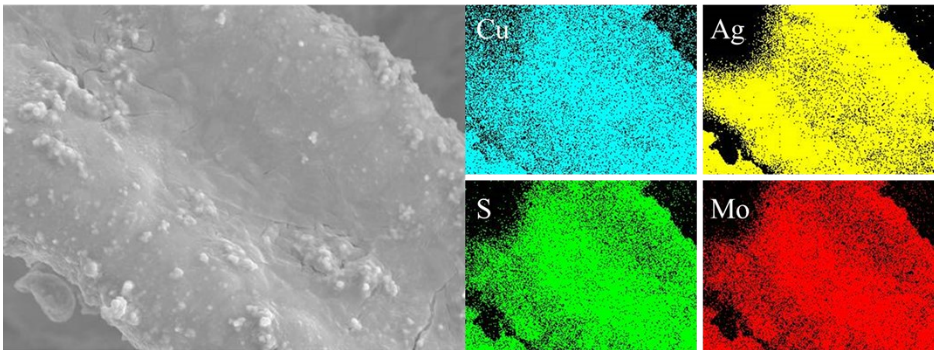

Figure S3. EDS mapping images of MoS_2@Ag-Cu foam after long-term cyclic testing.

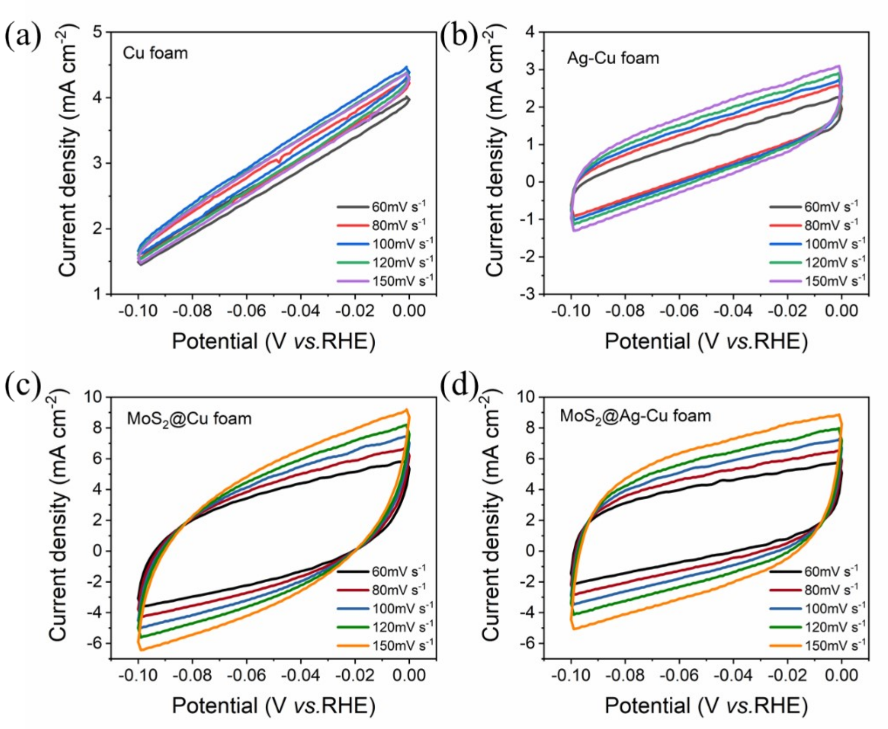

Figure S4. Electrochemical double-layer capacitance (EDLC) measurements of (a) Cu foam, (b) Ag-Cu foam, (c) MoS_2@Cu foam and (d) MoS_2@Ag-Cu foam, respectively. The current densities at 0.05 V vs. RHE and the corresponding scanning rates were used to calculate the EDLC value.

Table S1 The values of photo response current (ΔJ), overpotentials (η), Tafel, ECSA for Cu foam, Ag-Cu foam, MoS_2@ Cu foam, and MoS_2@Ag-Cu foam.

Samples / Values	Cu foam	Ag-Cu foam	MoS_2@ Cu foam	MoS_2@Ag-Cu foam
ΔJ (mA cm^2, Light/Dark) · Fig.4b	0.091	0.143	0.137	0.262
η (*vs.* 10mA cm^{-2}, V) · Fig.6a	0.370	0.278	0.223	0.218
η (*vs.* 50mA cm^{-2}, V) · Fig.6a	0.468	0.353	0.270	0.253
Tafel (mV dec^{-1}) · Fig.6b	153	115	68.5	54.2
ECSA (mF) · Fig.6c	1.8	11.2	38.5	53.2
